# Holistic Approach to a Successful Market Implementation of Active and Intelligent Food Packaging

**DOI:** 10.3390/foods10020465

**Published:** 2021-02-20

**Authors:** Sanne Tiekstra, Ana Dopico-Parada, Hanna Koivula, Johanna Lahti, Mieke Buntinx

**Affiliations:** 1 Bumaga BV, 6825 BS Arnhem, The Netherlands; sanne.tiekstra@huhtamaki.com; 2 Huhtamaki Molded Fiber Technology BV, 8938 AN Leeuwarden, The Netherlands; 3 Department of Business Organisation and Marketing, Universidade de Vigo, 36310 Vigo, Spain; adopico@uvigo.es; 4 Department of Food and Nutrition, University of Helsinki, 00790 Helsinki, Finland; hanna.m.koivula@helsinki.fi; 5 Materials Science and Environmental Engineering, Paper Converting and Packaging Technology, Tampere University, 33720 Tampere, Finland; johanna.lahti@tuni.fi; 6 Institute for Materials Research IMO-IMOMEC, Materials and Packaging Research & Services, University of Hasselt, 3500 Hasselt, Belgium

**Keywords:** active packaging, intelligent packaging, bioeconomy, sustainable food packaging, fiber-based packaging, socioeconomic roadmap, food packaging value chain, consumer’s perception

## Abstract

Market implementation of active and intelligent packaging (AIP) technologies specifically for fiber-based food packaging can be hindered by various factors. This paper highlights those from a social, economic, environmental, and legislative point of view, and elaborates upon the following aspects mainly related to interactions among food packaging value chain stakeholders: (i) market drivers that affect developments, (ii) the gap between science and industry, (iii) the gap between legislation and practice, (iv) cooperation between the producing stakeholders within the value chain, and (v) the gap between the industry and consumers. We perceive these as the most influential aspects in successful market implementation at a socioeconomic level. The findings are supported by results from quantitative studies analyzing consumer buying expectations about active and intelligent packaging (value perception of packaging functions, intentions to purchase AIP, and willingness to pay more) executed in 16 European countries. Finally, in this paper, we discuss approaches that could direct future activities in the field towards industrial implementation.

## 1. Introduction

Without packaging, many food products lose their distinctive characteristics or nutritional components rapidly. Next to quality assurance, food packaging communicates with the consumer by informing them about the content. Steered by the current mindset and requirements of consumers as well as emerging legislation, the food and packaging industries are very aware of the public focus on packaging and its impact on the environment. International policies act as strong drivers of development. The Sustainable Development Goals function as the blueprint to achieve a more sustainable future and address global challenges [1], and the European Commission targets bioeconomy including circular economy in its research agenda [2].

Food packaging plays an important role in lowering the environmental impacts caused by waste and carbon dioxide (CO_2_) [3]. Over the last 60 years, society has created 8.3 billion metric tons of waste; 76% of this waste is plastic of which 90.5 percent is not recycled [4]. Since 39.9% of European plastic demand is generated by the packaging sector [5], the sector wants to be part of the solution and focuses more on circular packaging loops with a global trend to address the plastic pollution/waste by targeting single-use plastics. The use of biobased materials in packaging decreases the dependence on fossil fuels [6]. Fiber-based packaging (potentially coated or combined with biobased polymers) offers emerging opportunities as a biobased alternative for plastic packaging with high recycling rates [7,8,9]. The use of compostable biodegradable materials could be encouraged and targeted especially for highly food-contaminated packaging [10].

Sustainability in food packaging includes material sustainability, i.e., packaging from renewable, recyclable or recycled materials, re-usable packaging, and reduced material use (light weight), but also requires that the packaging guarantees functionality, high product quality and food safety, and minimizes the avoidable food loss and food waste.

**Figure 1:**
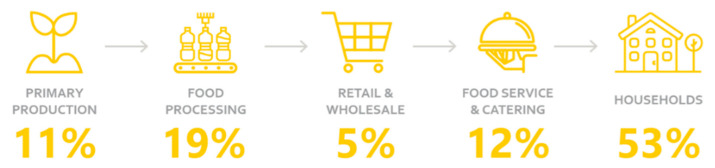
Food loss and food waste throughout the value chain (source: active and intelligent packaging in relation to food waste [12]).

Across the food value chain, 33% of the worldwide produced food is never eaten. According to the World Health Organization, this amounts to 1.3 billion tons globally per year, equalling 170 million ton of CO_2_ emissions [11]. The share of ”food loss” during the production, postharvest, and processing stages of the supply chain represents about 30%, whereas the loss of edible food occurring at the end of the food chain, i.e., during retail or final consumption, typically defined as ”food waste”, represents approximately 70%. More detailed shares of food loss and food waste occurring in each step of the food value chain are shown in Figure 1.

**Figure 2:**
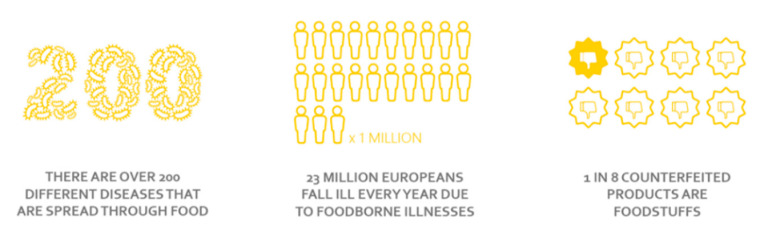
Concerns about food quality and food safety (source, AIP in relation to food safety [17]).

Food safety is, together with country of origin, cost and taste, the most important factor for Europeans when buying food. Around one in five Europeans says that food safety is their main concern when choosing food and. for two in five Europeans, it is among their concerns [13]. European consumers worry about the quality and freshness of food, about food poisoning, additives, and preservatives. This concern is supported by the fact that over 200 diseases are related to food-borne illnesses. These affect one in ten people and kill around 420,000 people every year worldwide as a consequence of the contamination of food by bacteria, viruses, parasites, chemicals, or toxins [14]. In Europe, every minute, 44 people fall sick from eating contaminated food, and almost 5000 people lose their lives every year [15]. Moreover, one in eight food products seem to be counterfeited. Although food fraud does not necessarily entail a risk to consumer health, it always implies a violation of the consumer’s interest to make informed choices and jeopardizes fair competition among enterprises (Figure 2) [16].

Innovative packaging concepts, more specifically active and intelligent packaging (AIP) concepts, can help to optimise the food supply chain, increase food shelf life and consumer consciousness of food utilisation, as well as introduce interaction between producer and consumer. Active packaging systems aim at maintaining or extending the product quality and shelf life by ”interacting” with the product, while intelligent packaging systems aim at communicating specific information and/or the quality of the packaged product during transportation and storage by ”monitoring” the condition of product [18,19,20].

### 1.1. Active Packaging Concepts for Shelf Life Prolongation

**Figure 3:**
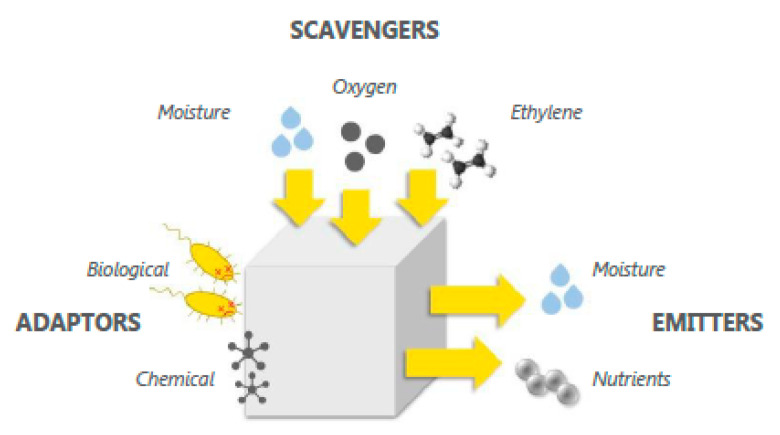
Schematic of concepts for prolonging shelf life (source, active packaging leaflet [25]).

The key function of food packaging is to protect products from changes in their environment, especially gases, ambient temperature, and relative humidity. Active packaging concepts interact with the packaged product or the atmosphere inside the packaging to ensure the quality, protect the valuable nutritional components, prevent quality degradation, and prolong shelf life. Depending on the type of food, however, quality degradation may be caused by different factors. Therefore, the following different types of solutions for prolonging shelf life have been created (schematically shown in Figure 3):

- Scavengers/absorbers are solutions that absorb substances from the packaging’s inner atmosphere (e.g., oxygen scavengers, moisture and ethylene absorbers) [21].
- Emitters are solutions that release substances into the packaging (e.g., CO_2_ emitters and antioxidants) [21].
- Adaptors are solutions that do not absorb or release substances but cause desirable chemical or biological changes in the packaged product or in the microbial life present in the packaged product or the packaging’s inner atmosphere (e.g., to reduce respiration or growth of microorganisms) [22,23,24].

Unfortunately, not all active packaging technologies that have been evaluated in model systems behave in the same way in real food applications. The complex structure of the food may influence the activity of the packaging, such as the release rates, absorption rates, or diffusion rates of active substances. Moreover, active substances or carriers may react with food components or bind to them, thereby, inhibiting the desired activity. Despite these challenges, numerous applications of product-tailored concepts achieving optimal activity of active packaging systems have been published [19] but only some have successfully made it to the market [21,26,27].

### 1.2. Intelligent Packaging Concepts for Advanced Communication

**Figure 4:**
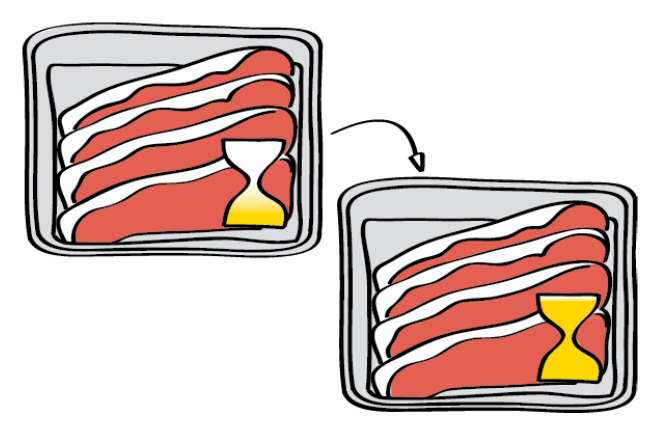
Schematic of an intelligent packaging concept with an indicator (source: intelligent packaging leaflet [33]).

In generic terms, intelligent packaging is used for communication as follows: (i) to inform about the product quality; (ii) to establish any brand-consumer connection; or (iii) to control tracking, theft, and counterfeiting conducts in the (food) supply chain [28,29]. The purpose of the first type of intelligent packaging is to monitor the environmental condition inside or in the vicinity of packaging, for example, gas production or consumption [30], relative humidity, temperature, or micro-organisms’ growth. Using this information, the quality of the packaged product and its shelf life can be estimated in order to guarantee its safety, for example, to inform the consumer about a break in the cold chain from packing to the consumers’ fridge. Secondly, intelligent packaging can play a major role in marketing and branding of a food product as packaging is the first thing that affects a consumer’s conscious decision to purchase a product. Interactive packaging can be a great tool to inform customers and to increase sales. Thirdly, the information flow within the food supply chain between producers, retailers, and consumers can be optimized using intelligent packaging. Intelligent packaging refers to a variety of technologies that are able to perform specific functions, and thus inform the consumer about the food quality and safety. In general, three types are differentiated according to their main function as follows:

- Indicators provide visual qualitative or semiquantitative information about the packaged food by means of a color change (e.g., different intensities or irreversible change) (Figure 4) [18,28].
- Sensors collect and provide quantitative information about the package and its content. They detect, record, and transmit information of the changes in the environment, the condition, or the operating history of the packaged food [18,31,32].
- Interactive packaging stores information regarding storage, distribution, and characteristics of the packaged food, in data carrier devices such as two-dimensional (2D) barcodes, radio frequency identification (RFID) and near field communication (NFC) tags, and electroluminescence displays. They enable more efficient information flow within the supply chain. It is also possible to integrate sensing materials with the data carriers to obtain information on the storage conditions (temperature and relative humidity) or food quality (microbial data) [28,30]. Intelligent packaging also paves the way to augmented reality in packaging to increase consumer engagement.

The proof-of-concept of various intelligent packaging systems has been widely reported in the literature and several technologies have made it to the market [31,34]. Opportunities that can drive the implementation of AIP include the Internet of Things (IoT) [35]. By 2025, not only mobile phones and computers, will be part of the IoT, but our homes and kitchens will also be part of the IoT, including food packaging (e.g., for tracking and tracing packaging equipped with sensors, actuators, or RFID tags) as part of this globally interconnected network infrastructure [36].

### 1.3. Challenges for Market Implementation of Active and Intelligent Food Packaging

Research and development in the field of AIP materials is very dynamic and develops in relation to the search for environment-friendly packaging solutions [37]. Since most of the current AIP solutions are plastic based, there is a clear demand for renewable and sustainable solutions to develop new fiber-based packaging materials with active and intelligent features. Subsequently, AIP technologies could balance or even counteract the current reality that fiber-based or renewable materials are often less effective barrier materials as compared with plastic-based food packaging solutions.

**Figure 5:**
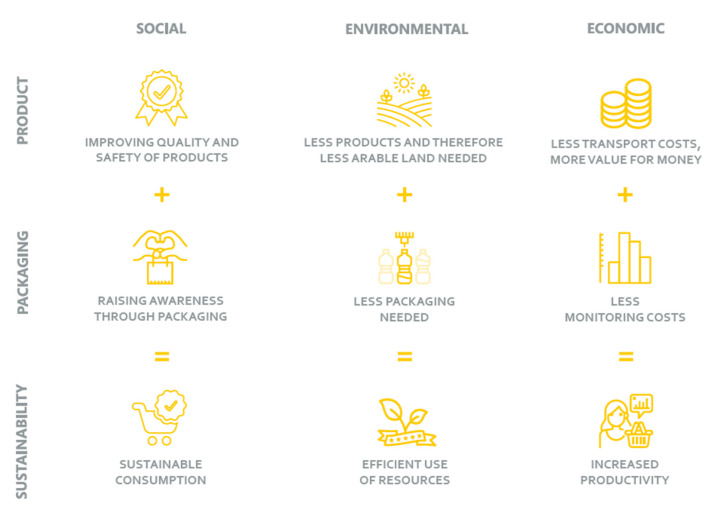
Summary of social, environmental, and economic impacts on sustainability (source, AIP in relation to sustainable development [38]).

A truly sustainable approach encompasses both the packaged product as well as the packaging (material). AIP can contribute to sustainable development in different ways, depending in which part of the packaging chain, interaction between packaging and product occurs. Three elements of sustainable development are of equal importance, i.e., economics, the environment, and social sustainability. The matrix in Figure 5 presents how these aspects interact. On the economic level, sustainable development includes food waste and losses, as well as material losses and disposal as wasted investments that might result in reduced incomes or increased expenses for customers. On the environmental level, less food waste and losses result in less need for arable land and reduction of unnecessary emissions. Next to improved ecosystems, this also leads to less need for packaging, which in turn results in less packaging disposal. On the social level, quality and safety of products can be improved by the use of AIP and awareness of the product, its quality, and disposal can be raised by packaging. Extended shelf life can also contribute to lower dependence on availability peaks leading to financial gains in the off-season. Sustainable consumption, efficient use of resources, and increased productivity could be reached and sustained in an efficient value chain when all above aspects are appropriately addressed.

Despite the invested effort and successful introduction of active packaging products in Japan and the USA, no such “boom” has been identified in the EU market. Whether it is a result of prohibitive legislation, lack of market demand, consumer wishes and needs, or lack of availability of technical solutions, the route forward remains unclear. As successful market implementation of active and intelligent packaging solutions in fiber-based industries is limited to date, this research gathers social and economic factors that can act as barriers towards market introduction. It is important to identify and understand the factors preventing developed solutions from reaching the market, which companies need to address before launching AIP products. In this paper, we discuss all elements required for a holistic approach to a successful market implementation of active and intelligent food packaging.

## 2. Materials and Methods

The basis for this study is the consolidated roadmap as delivered by COST Action FP1405 ActInPak (2015–2019) [20,39,40]. In this network, over 400 participants from research organizations, industry, and branch organizations/policymakers from 34 European countries in the areas of papermaking, printing, packaging, bio-based materials, and chemicals openly discussed implementation of AIP. These open discussions, workshops, and conferences resulted in a vast amount of scientific and technical literature being collected and shared and, in several roadmaps directing future activities in the field. In addition to this qualitative exploration, a quantitative consumer survey was conducted into the current value perception of packaging functions, intentions to purchase AIP, and the willingness of European millennials (Gen Y) to buy AIP.

### 2.1. Socioeconomic Roadmap for Implementation of Active and Intelligent Packaging (AIP)

**Figure 6:**
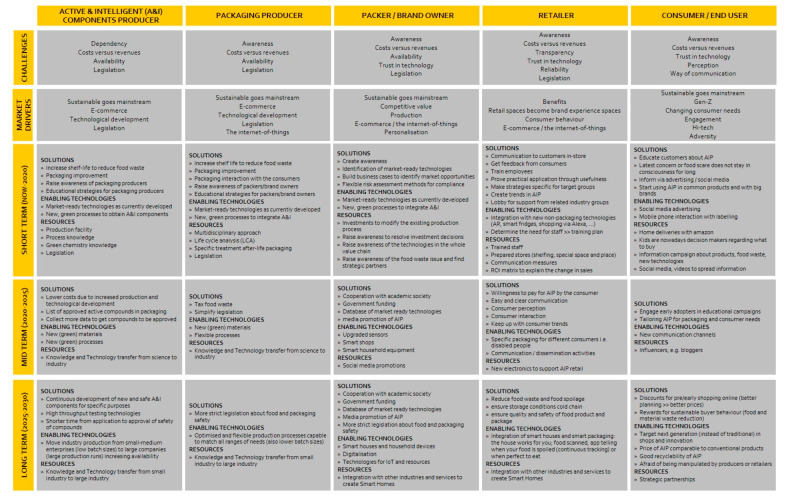
Socioeconomic roadmap for Active and Intelligent Packaging (AIP) composed by Working Group 2 of COST Action FP1405 ([41], Supplementary Material).

Roadmap activities were performed over the course of four years, with two to four meetings per year. Attendees of these meetings and dedicated workshops were technologists, industry experts coming from all areas of the value chain, and market or consumer experts, forming multidisciplinary working groups. During the roadmap workshops, challenges were discussed using various problem-solving methods and brainstorming techniques. Input was also provided by conferences, scientific literature reviews, research cooperation, and cooperation with industry associations. Through these efforts, information, trends, and activities were gathered, followed by identifying priorities and relations in the value chain. Results were discussed in order to define obstacles and implementable strategies and actions for efficient industrialization. The created roadmap encompasses challenges and market drivers that affect the involved value chain stakeholders. Additionally, solutions, enabling technologies and resources to aid successful market implementation for short term, mid-term, and long-term approaches were specified. In this paper, we share the socioeconomic roadmap (Figure 6), which addresses aspects relating to the interaction of social and economic factors [41]. Other roadmaps that were created focused on technical challenges and sustainability aspects [40].

### 2.2. Survey about Consumer Buying Expectations of AIP

An online survey was conducted to validate the qualitative findings of the abovementioned roadmap sessions. The online survey, addressed to European consumers, was conducted in 2019. The aim was to determine the consumer buying expectations of active and intelligent packaging (value, purchase intention, and willingness to pay more). The sample for this study was comprised of 1249 individuals from the following 16 European countries: Bulgaria, Finland, France, Germany, Italy, Netherlands, Norway, Poland, Portugal, Romania, Russia, Serbia, Slovakia, Slovenia, Spain, and the UK. Respondent demographic characteristics are presented in Table 1. The majority (64%) of the respondents were female, 29.7% of all respondents lived alone or with another person, and 11.4% had children. All of the respondents were born between 1980 and 2000. This age group (Gen Y), which accounts for 9.5% of the total population in Europe, was chosen because they represent current and potential future buyers of AIP. Data were summarized as frequencies for each question and presented in contingency tables. In addition, significance was determined using chi-square analysis in IBM SPSS software (version 23.0, Vigo, Spain).

## 3. Results and Discussion

### 3.1. Socioeconomic Barriers towards Implementation of AIP

In this paper, we do not discuss AIP technologies for food products, but specifically address social and economic interaction factors between the AIP industry (that needs to implement AIP) and consumers (that use AIP). On the basis of the socioeconomic roadmap generated by the ActInPak network (Figure 6) [41], we studied how the different food value chain stakeholders with different interests and prospects could direct future activities towards successful market implementation of AIP. The identified factors include the following: (i) market drivers that affect developments, (ii) the gap between science and industry, (iii) the gap between legislation and practice, (iv) cooperation between the producing stakeholders within the value chain, and (v) the gap between an industry and consumers.

#### 3.1.1. Market Drivers Affecting Developments

To align with overarching strategies as mentioned in the introduction, the main trend in material development is sustainability (including circular and bioeconomy). Many brand owners and food manufacturers position sustainability as a key factor in their strategy, evaluating their options for packaging materials and solutions from this perspective. Reduction in material, in general, is critical for packaging manufacturers and there is a clear shift from plastic materials to paper-based solutions with enhanced barrier properties where possible [42,43,44]. In addition, consumer awareness is continually increasing. As witnessed by current events such as climate marches, they demand more sustainable solutions. This results in initiatives towards no plastic (alternative materials), no waste (reusable packaging materials), or even no packaging (“naked” products on the shelves) with increasing followers and success [37].

In the paper and board industry, various stakeholders such as the paper producers and their raw material suppliers are developing materials that have similar or improved characteristics as compared with plastics to act as a replacement for nonrenewable materials [45,46]. Throughout the food packaging industry, sustainability is at the top of the agenda, and whilst preserving shelf life is critical, there is a focus to simplify multilayer packaging and drive monomaterial solutions [47]. Obviously, food producers, brand owners, and retailers do not want any compromise on shelf life due to food waste concerns. The development of novel barrier materials is in a stage where they maintain the quality of the food and prevent food waste better than current paper-based packaging materials. As improving shelf life is the main aim of active packaging, one could argue that the need for active packaging materials disappears if the protective function has already been fulfilled by incorporation of barrier layers (e.g., coatings, and laminates). However, active components have the potential to reduce the numbers of layers needed to reach the required barrier properties. Since the fiber-based sector commonly focuses on development of barrier materials to increase their competitiveness compared to the plastic packaging industry, those barrier materials are often cheaper or at least easier to finance than incorporating additional novel features. In most cases, infrastructure and processes of packaging production need to be adapted to be able to work with active and intelligent materials. For example, for printed electronics, implementation in roll-to-roll processes will need more changes. Nevertheless, the applicability of new materials is very food category dependent, and the fundamental difficulty of packing high moisture content food for long-term storage using only biobased materials could still be aided by introducing AIP technologies.

For intelligent packaging, the situation is different, since the application areas seem to increase. Market drivers such as the Internet of Things (IoT) are easy to address with intelligent packaging features [35]. These could allow creation of advanced e-commerce shopping experiences where the packaging in the fridge informs an order system about the need to repurchase so the consumer does not even have to think about it. In addition, traceability improves, as the product can be better tracked, linking the food value chain parties and the consumer, and monitoring its quality and the way it is handled or used. For short-term products or single use take away, intelligent packaging might have an added value in, for example, proving that the temperature of the food has not decreased below preferred temperature. Additionally, intelligent packaging features can offer brand owners interesting opportunities through consumer engagement and data collection [48].

In AIP, consumer behavior, changing needs, and consumer acceptance play very important roles for driving innovations. New generations (e.g., Gen Y and Gen Z) have different expectations from global brand owners and are inclined to switch to small innovative players if they answer to their wishes and needs, whereas older generations seem to be more loyal to established brands that they are familiar with. For sustainable market success, these drivers are indispensable for understanding how to avoid adversity of products and brands.

Recently, the global COVID-19 situation has been perceived to be a new market driver. Many companies commenced exploring antiviral packaging solutions in various stages of the value chain. The solutions could possibly reduce (cross) contamination during production and use situations and provide safety for customers. For example, antimicrobial pharmaceutical packaging can prevent cross contamination amongst hospital departments and coated cardboard boxes can ensure a product is transported from supplier to recipient without contamination. However, the gap between science and industry, which is discussed next, becomes very apparent in the current situation. Some companies have experienced confusion, for example, between the terms antiviral and antibacterial solutions, and they lack clear communication about the effectiveness of current solutions and safety of materials [49]. They need effective transfer of technologies into their applications, and input of testing labs to verify their concepts (e.g., are substances heat resistant in order not to lose effectiveness in a hot production process?).

#### 3.1.2. Gap between Science and Industry

Although the term active packaging was introduced more than 30 years ago and numerous scientific publications in this field are available [50,51], the term is quite new to industry in general and relatively few applications can be found in the market [21,26]. One example, in the market, demonstrates how anti-spoiling and freshness-preserving properties can be added to extend the shelf life of fruit by applying a sticker containing a formulation with natural ionized salt [52,53]. In addition, interest in nanocellulose-based packaging applications is accelerating rapidly. Promising strategies for enhancing barrier and mechanical properties using nanocellulose have been demonstrated or are in different stages of implementation. By using nanocellulose, fiber-based packaging can be given antibacterial properties without compromising its sustainable properties [54]. However, the best choice of material composition and material processing, for example, melt-extrusion or electrospinning technologies for efficient incorporation into packaging products, requires more research in the coming years to achieve the full potential of nanocellulose in a wide range of packaging applications [55,56].

The use of nanotechnology is likely to be very important for the development of AIP [57,58]. However, the benefits of engineered nanoparticles in food contact applications are accompanied by safety concerns, due to gaps in understanding their possible toxicology if consumers are exposed as a result of potential migration of the nanoparticles from the material into food [57]. Therefore, in the European Union (EU), this technology is only authorized under strict conditions set out by the member states and the European Commission after case-by case risk assessments by the European Food Safety Authority (EFSA) (see further in Section 3.1.3).

There is a lack of awareness and knowledge about AIP, its benefits, added value, function, and impact [59,60]. In hectic business life, there is a lack of time as well. Even though widely available, scientific publications do not necessarily help in this regard. Communication targeted towards industry should include useful information in a format that can be processed in short periods of time, i.e., a type of sales pitch, short and with relevant, concrete examples. Information should not focus on partial solutions, but rather provide the big picture. Communication should describe the product, how it can be safely used by the company, how much it will cost, and information that it complies with legislation, etc. Communications should be related to situations that also show the different scenarios and targeted applications (e.g., bulk versus unit, or high value specialty products). When presenting a new AIP solution, it would be good to have a “proven” demonstrator to show the potential of the solution [60]. Furthermore, it is important to discuss opportunities for AIP technologies with different people inside companies, not only engineers but also marketing and design departments as well as purchasing departments should be part of the discussions to involve the whole value chain.

Another gap to overcome is the move from lab to industrial scale. According to lab scale results, expectations for effectiveness of active components in real-life conditions are often too high. The real food product, storage conditions, process conditions (e.g., mechanical characteristics, chemical changes of coatings, and sealing properties), and material characteristics, all influence the effectiveness [21]. Successful transfer from application to industry is very sophisticated. Before industrial implementation is possible, food approval or even biocide approval might be needed. The approvals take time, whereas time is often of essence for market opportunities to succeed. This is the reason why development and challenge tests under real conditions are often done by publicly funded research projects, as companies do not have time for this. However, a research project is often dedicated to a specific application and not transferable to a wider application. In addition, usually the production and packaging machines dictate which AIP technologies are possible to upscale (or not).

#### 3.1.3. The Gap between Legislation and Practice

In the EU, AIP for food applications must comply with the European Framework Regulation (EC) No. 1935/2004 and if made from plastic materials with Regulation (EU) No. 10/2011 on Food Contact Materials. It states that materials and articles, including active and intelligent materials and articles shall be manufactured in compliance with good manufacturing practices (Regulation (EC) No. 2023/2006) so that, under normal or foreseeable conditions of use, they do not transfer their constituents to food in quantities which could (i) endanger human health, or (ii) bring about an unacceptable change in the composition of the food, or (iii) bring about a deterioration in the organoleptic characteristics thereof. Due to the deliberate interaction of AIP with the food or its environment, the migration of substances could represent a food safety concern [61]. Therefore, special requirements for active and intelligent materials and articles are included in Regulation No. 1935/2004.

More specifically, Commission Regulation No. 450/2009 states that the individual substances, which make up the active or intelligent component, should be safe and comply with the requirements of the framework regulation No. 1935/2004. The safety of active substances present in packaging must be evaluated by the EFSA before their possible inclusion into a positive Community list. Only individual substances and combinations of substances assessed by EFSA can be used in components of active and intelligent materials and articles. However, the EU Guidance document shows that suppliers, while in the process of having their active packaging approved, may place AIP solutions on the market, provided all other applicable European and national regulations are complied with, demonstrating that they do not present risks to human health. From the date of application of the Community list, only packaging that complies with compositional requirements can be marketed [62]. The regulation also specifies that active and intelligent materials should be labelled as nonedible to avoid accidental consumption and that information should be provided throughout the package chain to ensure their correct use. These regulations are quite extensive and, often, it is not clear to industry where to consult or what to refer to, in order to ensure that the product entering the market is food safe. The approval processes take quite a long time, whereas companies’ market introduction processes do not account for these lengthy routes. While following the required procedures, companies can miss a window of opportunity in the market, or possibly take a considered risk with bringing a product to the market that may affect consumer’s health and safety and result in economic risks of recalls, claims, and reputation [63].

#### 3.1.4. Cooperation between the Producing Stakeholders within the Value Chain

It is very important to identify the entire value chain of a particular product and to identify who the key decision makers are [64]. The identified value chain runs from active and intelligent component producers, via packaging producers to packers, brand owners and retailers, to the end user. The identified challenges affecting successful market implementation of AIP, are rarely an issue of one stakeholder; they run throughout the value chain and influence multiple stakeholders. Basically, there is an interconnection between the stakeholders in the form of mutual challenges, even though the details of the challenges differ. Most recurring challenges trace back to cooperation and communication on different levels within the value chain, as well as intersectoral challenges. Many of the challenges, shown in Figure 6, could be overcome by increasing communication and cooperation between stakeholders within the same value chain as well as stakeholders in different sectors. Conflicting interests can easily create boundaries that seem to halt collaboration.

An example of this is a challenge such as costs versus revenues. It is clear that for some stakeholders this means they must obtain more value for investing money in novel, more expensive materials [46,65]. A value chain approach argues that the benefit of active packaging can be found in reduced food waste, and for intelligent packaging, the benefit lies in (better) verified safety or interaction between different parts of the value chain. One could argue that if waste is reduced, fewer products need to enter the market, and therefore fewer products need to be produced and packaged. However, since the actual profit of reduced food waste occurs elsewhere in the value chain, the packaging producer and the brand owner are only affected by lower sales, and thus lower profits due to lower production needs. This could be overcome by cooperative agreements or even with appropriate marketing to increase product prices and to make up for the losses [3,64].

Another example underling the need for good cooperation within the value chain is the challenge of availability, where production size does not match demand or where materials cannot be further developed or adjusted to unknown needs as behavior and interaction are not yet known. Currently, this challenge creates a vicious circle among different stakeholders; AIP component producers, at first, are not able to produce tons of their components as they wish to validate them first. In turn, packaging producers are often not capable of running small validation trials or are not willing to run a trial without clear proof that there is a market for it. Yet, to prove the market potential a trial is needed [65].

#### 3.1.5. Gap between Industry and Consumer

Once active or intelligent features in food packaging have been successfully incorporated, one would think it is an automatic success in the market. However, it has been clearly identified that there is currently a gap between industry and consumers which hinders a successful market introduction [65,66,67,68,69,70].

Consumers do not recognise shelf life-prolonging packaging. Most consumers do not even realise that some of the solutions they are using on a daily basis are in essence prolonging shelf live, such as modified atmosphere packaging (MAP). This might actually be a reason why MAP is widely accepted on the market. The fact that emitters and absorbers, and antimicrobial components are unknown to consumers and that they would be perceived as an additional component of the packaging, may be the reason to reject active food packaging, reflecting a lack of awareness and trust in the technology. Consumer education and knowledge building is needed, especially in these days of information overflow and false new where awareness and opinions are more prominently present in consumers’ truths. It is important that the AIP features are not too complicated for a consumer to understand; there should be no room for misunderstanding [71]. However, the way in which information is presented also affects consumer’s trust [72]. For example, an unknown and invisible extra feature in packaging (e.g., an active barrier coating that releases substances to the packaged food), is perceived to be suspicious when the benefits are unknown and unclear. As a result, consumers may doubt the safety and healthiness of the packaging and turn to alternatives.

The concept of freshness is challenging and tricky in combination with extended shelf life. Perceptions of freshness deeply rooted and vary across consumers, countries, and cultures, even in products that are already familiar. If the perception of freshness influences the perception of healthiness of frozen and canned products versus fresh produce, extended shelf life could be perceived as less fresh or artificial. However, if communication clearly stresses the fact that through (completely food safe) active packaging the food is more natural because chemical preservatives are omitted, consumers might be inclined to pay the extra cost [73].

One could also argue the extent to which a consumer has blind faith in technology, or whether a consumer uses their own knowledge and common sense to assess the quality and safety of food products. When freshness indicators are introduced to communicate about the quality of a food product, there is a risk that consumers could rely solely on these indicators [74]. On the one hand, this can be positive when the packaging indicates the food is still safe to consume after the expiry date and can be consumed rather than considered to be spoiled. On the other hand, when an indicator shows an earlier expiration than the printed expiration date it can encourage and justify turning the product to waste. Studies have also shown that the return rate of perceived “bad” products increased [74]. Consumers were inclined to return more quickly to the supermarket for a refund because the indicator showed the freshness of their product might have been affected. Whether this was due to a compromised cold chain, or due to the influence of warm hands taking the product out of the bag and into the fridge at home, the consumer or the retailer could not possibly know. Without an indicator, consumers use their own awareness to assess the quality and freshness of products and only turn to companies when something is wrong before the expiration date. However, indicators do prove their value in secondary or tertiary packaging during logistics (e.g., food storage, transport, and home delivery) by ensuring uncompromised cold chains.

**Figure 7:**
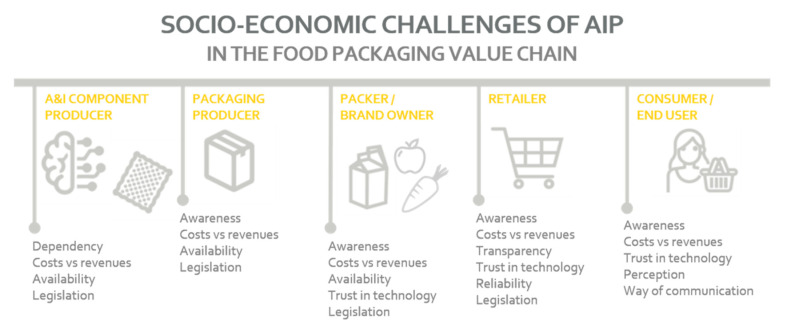
Overview of socioeconomic challenges for all different stakeholders in the AIP value chain [41].

A summary of the discussed socioeconomic challenges that can hinder the implementation of AIP from the viewpoint of different food packaging value chain stakeholders is shown in Figure 7.

### 3.2. European Consumer Perception of AIP

#### 3.2.1. Value Perception of Packaging Functions

To validate the findings, Gen Y respondents were chosen because they represent an important segment of the population. Some of them are so economically valuable that advertisers abandon their existing methods to cater to them. Others are likely to get richer over time and represent an important potential market for advertisers and consumer companies. Consumers’ value perceptions about traditional and new packaging functions were scored on a scale of seven points, where 1 represents “not important” and 7 “very important” (Table 2). The overall value of packaging was clearly appreciated as important (score 5.3), as were the following different dimensions: protection (6.0), sustainability and economy (5.8), information (5.5), convenience (5.2), portability and storage (4.8), and promotion (4.0).

The results confirm that the main function of packaging is to protect a product. This is in line with other empirical data showing that the protective function of packaging is a quality that is considered to be a “must”, especially for food products [75,76,77,78,79]. This also links well with the second most important function of packaging for consumers, i.e., sustainability [80]. Our results indicate that young European consumers (in 2019) value packaging that offers sustainable solutions with regard to rational use of resources, environmental impact, and social demands such as accessibility. This was also observed by Boz et al. [37], in contrast to earlier studies that indicated consumers valued other aspects more than sustainability [81,82,83]. With an equal level of importance, price is a relevant aspect of packaging for consumers. As stated by Nordin and Selke [84], efforts to ensure that packaging fulfils its functions must be made in balance with consumer price sensitivity.

Consumers in this study granted the next level of importance to the informational function of packaging (e.g., information about content, benefits, legal regulations, brand value, and technical matters relating to intelligent packaging). This result is in line with other empirical studies that have stated that informational elements are factors that significantly influence the opinion of consumers buying a food product. They might even be more important than visual and other promotional elements [79,83,85,86]. The next function of packaging, in order of importance according to the surveyed consumers, is convenience. Another study has also indicated that consumers consider convenience as an important modern feature of packaging due to changing lifestyles and that handiness of packaging is crucial in consumer food packaging choices [87], although this study was not applied to food products in general but to soft drinks, for which portability is highly valued by consumers. Convenience is followed by the portability and storage function. This means that facilitation of the consumption of the product anywhere and storage in the home is less important for consumers than other functions.

Finally, consumers value the promotional function of packaging such as design, color, light, sound, and games. This consideration has been reinforced in previous studies that focused on other actors in the value chain as marketing managers. They usually pointed to packaging as a key marketing tool within a highly competitive food industry [83,88]. Definitely, this function has the ability to attract customer attention and enhance a product’s image, although consumers only moderately value this function.

Some questions specifically surveyed the importance of AIP functions for consumers. The answers indicated that consumers value active packaging (AP) functions (5.9) more than intelligent packaging (IP) functions (4.8). With regard to AP, consumers consider safety quality control functions more important than those that provide freshness or enable extended product life. In the case of IP, functions related to information about freshness, authenticity, and temperature proved to be more important than functions with interactive properties (sensorial or leisure experiences).

#### 3.2.2. Purchase Intention of AIP

Regarding the purchase of AP, consumers report that they would buy packaging containing papers or films with antioxidant or antimicrobial properties (5.0), packaging incorporating pads in order to absorb excess liquid and release substances that improve the preservation of the product (4.8), packaging incorporating natural additives that reduce oxygen intake and gas emission by extending the life of the product (4.6), and packages that present a “ready to drink or eat” format (4.0) (Table 3).

For IP, respondents report that they would buy packaging that indicates a product has been maintained at the correct temperature (5.9), indicates a product is at the ideal consumption temperature (5.5), or interacts with the brand and the product (4.7). However, packaging that interacts by providing engagement and leisure is less appreciated by the respondents (e.g., virtual reality, conductive inks, and QR codes) (4.0). Finally, the purchase intention is lowest for packaging that emits flavor (3.8), light, or sound (2.7) (Table 3).

Table 3 shows that habitat, household with/without children, and gender are also factors that influence the intention to purchase AIP. According to a cross tabulation analysis, there are some differences between urban and rural populations with respect to purchase intention of AIP. While consumers from urban areas show a slightly higher purchase intention for active packaging, consumers living in rural areas show preference for intelligent packaging.

In addition, the presence of children within the household is related to a greater purchase intention for the AIP tested. Related to gender, although the grade in which men and women develop the purchase intention is quite similar, men show a little more propensity to purchase AP and women show some preferences on IP especially those that provide information. In line with other studies, we argue that it is possible that women had a more careful approach to new technologies because of their role as a nurturer and care provider in the family, while they prefer to monitor the history, freshness, and quality of food [77,89]. Moreover, an independent t-test comparing means between samples explains the significant differences (*p*-value) for each type of packaging (e.g., ready to eat/drink packaging are preferred by urban men in families with children and packaging with lights and sounds develops a greater purchase intention among families with children living in the rural area).

#### 3.2.3. Willingness to Pay Extra for AIP

Previous studies have highlighted that consumers have a positive attitude and are willing to pay more for some AIP [75,77,90,91]. Most of these studies only focused on consumer acceptance of oxygen absorbers or time-temperature indicators. The present study also included the acceptance of other types of AP such as emitters or adaptors and other IP such as toxin indicators, packaging with sensors or interactive properties (e.g., three-dimensional (3D), virtual reality, and games).

**Figure 8:**
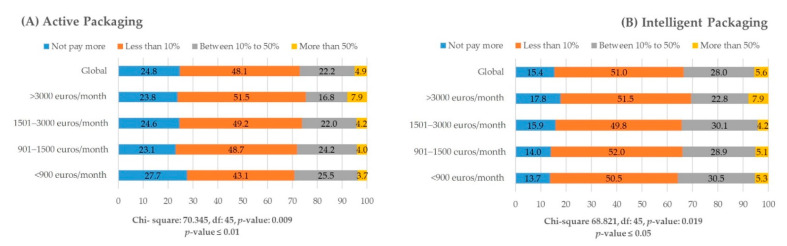
Willingness to pay more. (**A**) For active packaging; (**B**) For intelligent packaging.

The results of this study indicate that the purchase of AP is dependent on the price. For active packaging, 75.2% of respondents are willing to pay somewhat more for packaging with active properties. However, 48.1% of respondents are only willing to pay less than 10% for AP. Only 22.2% of respondents declare their willingness to pay more than 10% and less than 50% for AP. Finally, 4.9% of respondents agreed to pay more than 50% for AP (Figure 8A).

The situation is better for IP. The vast majority of respondents (84.6%) indicated they would be willing to pay an extra price for a packaging with intelligent characteristics as opposed to normal packaging, 51.0% of respondents would only be willing to pay less than 10% more for a product, 28.0% of respondents would pay up to 50% more, and only 5.6% of respondents would pay more than 50% (Figure 8B).

The analysis of the relationship between family income and willingness to pay more for AIP showed that although families with the lowest family income are less willing to pay more, this intention does not increase as income increases. The chi-square test of independence was used to determine significant associations between family income and willingness to pay more for active (*p* < 0.05) and intelligent packaging (*p* < 0.01) (Figure 8A,B).

## 4. Conclusions and Recommendations for Future Research

It is tempting to make generalized recommendations for AIP; however, viewed from a socioeconomic market implementation perspective, active (shelf prolongation) and intelligent (communication) technologies for food packaging have different purposes with different implications. Similar to all packaging developments, market drivers also affect AIP developments. Market drivers offer many opportunities with the growing digitalization relating to, for example, product safety, product location and product loss, combined with increased demand for sustainable solutions. The IoT can drive the implementation of AIP. The next step in digitization after “monitoring” should be to manage and control the conditions of goods in real time. Despite these emerging technologies, the full potential of intelligent packaging as one of the most exciting application domains of Industry 4.0 can only be reached if cybersecurity is appropriately addressed [35].

Novel active packaging solutions have proven to be more difficult (as compared with intelligent solutions) to implement in business to consumer environments for multiple reasons including incorporation in current infrastructures, the costs of investment, the different stakeholders with different benefits, and the consumer acceptance (Figure 8A,B). More importantly, it is a question whether active packaging developments are still spot on when looking at current market drivers and global megatrends. This does not exclude the fact that active features are beneficial to add on top of the need for improved quality (which can also be reached by novel barrier materials), for instance, in cases where preservatives are added to the packaging material instead of to the food itself, so the packaged food can be as natural as possible.

The recent global COVID-19 pandemic has given companies reasons to explore the introduction of antiviral solutions at various stages in the value chain. However, confusion, unclear communication about effectiveness of current solutions, difficulties in transfer of technologies into their applications, access to testing labs to verify concepts, among others, make drafting up a viable business case a challenge in itself.

Concerning the gaps between the different value chain stakeholders, i.e., science versus industry and industry versus consumers, we conclude that the brand-consumer connection is already widely used in the market, and therefore the challenges are relatively low. The reason for this is that most commonly used intelligence features are labels, and thus add-ons to a packaging, and therefore there is less need to change the current infrastructure or processes. Furthermore, extra costs for these features are often paid by marketing budgets and they pay off via consumer engagement and repeated buys. Consumers buy this type of packaging because they like it. The informing aspect, where the focus is more related to product quality and less to consumer engagement, is more difficult to implement. The main reason for this is the fact that these features try to make the value chain transparent for the consumers by informing them whether the quality of the product was compromised or not. These products might lead to profit losses at retailers, causing fear to use it or potentially force them to pay more attention to shelf circulation and stock management. It might also result in fear at the consumer level, instead of grown confidence.

To reach consumer acceptance of AIP in food packaging, more research is needed to explore the perception of different consumer groups, such as the elderly [38] or the next generations (e.g., Gen Z), in addition to the surveyed European generation Y. Different expectations, as well as cultural differences are relevant for future acceptance of active and intelligent food packaging.

Although the focus was mainly on the implementation of AIP for food applications, AIP can also prevent waste by improving vase life for flowers and guarantee the quality of high value products such as electronics or cosmetics and the authenticity of medicines [20]. For pharmaceuticals and high-tech products, more money is available for research and development; this combined with an often-smaller scale (as compared with food products) enables lower risk market introduction of novel technologies. Additionally, in pharmaceuticals, legislation is stricter as compared with food, and thus might be a bottleneck for new developments. Legislation by itself is a topic to consider on different levels; at the industry level, regulations and their implications may not be completely clear, whereas at the policy level, the approach of AIP legislation (i.e., approval of substances and packaging solutions) may need to be better streamlined to make AIP implementation more attractive and easier to implement.

Overall, for a successful market implementation of AIP, a holistic view should be taken, including technological, sustainability, and socioeconomic aspects, and a value chain approach should be applied. With regard to the cooperation between the producing stakeholders within the value chain, the global COVID-19 pandemic has shown that companies are able to cooperate along the value chain and to decrease lead times to market solutions in the urgent search for antiviral packaging. However, if communication about possible AIP components, their effectiveness, and implementation is clearer, this challenge would be easier for industry.

## Figures and Tables

**Table 1:** Sociodemographic characteristics of the sample.

Variable	*N* = 1249	
	*N*	%
**Sex**		
Male	449	35.9
Female	800	64.1
**Home**		
Single family home	88	7.0
2 People	283	22.7
3 People	382	30.6
4 People or more	496	39.7
No children at home	1107	88.6
Yes, children at home	142	11.4
**Situation**		
Employee	374	29.9
Study and employed	270	21.6
Unemployed	61	5.1
Study	542	43.4
**Habitat**		
Rural	366	29.3
Urban	883	70.7
**Income**		
Less than 900 euros/month	346	27.7
Between 901 and 1500 euros/month	279	22.3
Between 1501 and 3000 euros/month	312	25.0
More than 3000 euros/month	102	8.2
Don’t know/Don’t answer	210	16.8

**Table 2:** Value perception of packaging functions.

	ITEMS	Mean		SD
	**1. PROTECTION**	**6.0**		1.0
	To prevent deterioration of the product	6.0		
	To prevent damage	5.3		
**ACTIVE**	To ensure the safety of ingredients and the product	6.1	**5.9**	
	To preserve product quality and taste of ingredients	6.2		
	To prevent contents from escaping or penetration by liquids, vapours	6.2		
	To provide antimicrobial properties that extend product life	5.4		
	To preserve hygiene by avoiding chemical interaction with the product	6.1		
	To maximize product freshness	5.9		
	**2. CONVENIENCE**	**5.2**		1.2
	To facilitate openability of the package	5.4		
	To facilitate product handling (ergonomic packaging and/or lid)	5.2		
	To provide a good system for closing the package	5.4		
	To ensure convenient, fast consumption of the product: cool, hot	4.7		
	Size in line with consumption needs	5.3		
	**3. PORTABILITY & STORAGE**	**4.8**		1.3
	To facilitate storage in the pantry, minimizing the need for space	4.7		
	To make the date of preferential consumption very visible	4.7		
	To decrease in size after partial consumption of the product	4.4		
	To make the product easy to carry	5.1		
	**4. INFORMATION**	**5.5**		1.1
	To report rigorously on its content	5.8		
	To indicate the benefits obtained from consumption of the product	5.5		
	To contain information on prices	5.1		
	To inform about legal regulations	4.8		
	To transmit brand’s ethical values	5.0		
**INTELLIGENT**	To report product temperature avoiding a break in the cold chain	5.1	**4.8**	
	To report the product origin	5.6		
	To guarantee product authenticity	5.7		
	To inform about food	6.0		
	**5. PROMOTION**	**4.0**		1.4
	To stimulate senses: emitting lights, sounds, voices, smells, etc.	3.4		
	To provide leisure experiences (games, QR-codes, 3D, virtual reality, etc.)	3.1		
	To provide suitable visual design (color, logo, graphics, size, etc.)	4.9		
	To provide entertainment and consumer fun	3.3		
	To contain information that helps guide decisions at the point of sale	5.2		
	To be innovative or different from other packaging	4.6		
	To offer an element to be kept or collected	3.9		
	**6. SUSTAINABILITY**	**5.8**		1.2
	To not contaminate the environment in its manufacturing process	5.8		
	To not waste resources in its manufacturing process	5.6		
	To facilitate access for people with visual impairment or handling difficulties	5.6		
	To be biodegradable	5.9		
	To be reusable, recyclable or returnable	6.0		
	To be rational with the use of resources, avoiding waste	6.0		
	**7. ECONOMY**	**5.8**		1.2
	To be low price	5.6		
	To offer good value	6.0		
	To not excessively increase the product price	5.9		
	**TOTAL**	**5.3**		0.9

7-point Likert scale (1, not important; 7, very important). Functions of packaging in bold. The gray background indicates active and intelligent packaging properties.

**Table 3:** Mean values (and standard deviations) of the respondents’ views on purchase intention of AIP.

		Habitat			Children at Home			Gender		
Would You Buy…?	Global	Rural	Urban	*p*	No	Yes	*p*	Men	Women	*p*
**ACTIVE**										
Packaging that presents a “ready to drink/eat” product	4.0 (1.83)	3.8 (1.81)	4.1 (1.82)	*	4.0 (1.79)	4.4 (1.99)	*	4.2 (1.72)	3.9 (1.86)	**
Packaging that contains pads to absorb the liquid waste and release substances that improve product conservation	4.8 (1.71)	4.8 (1.78)	4.9 (1.68)		4.8 (1.71)	5.1 (1.68)		4.9 (1.70)	4.8 (1.73)	
Packaging that incorporates natural additives that reduce oxygen and emission of gases extending the life of the product	4.6 (1.81)	4.4 (1.87)	4.6 (1.79)	*	4.5 (1.81)	4.9 (1.81)	*	4.7 (1.76)	4.5 (1.84)	*
Packages with papers or films with antioxidant properties and/or antimicrobial agents	5.0 (1.60)	5.0 (1.62)	5.0 (1.60)		4.9 (1.59)	5.2 (1.66)		5.1 (1.49)	4.9 (1.66)	
**Total**	**4.6**	**4.5**	**4.7**		**4.6**	**4.9**		**4.7**	**4.5**	
**INTELLIGENT**										
Packages with lights and/or sounds	2. 7 (1.73)	2.9 (1.79)	2.6 (1.71)	*	2.6 (1.68)	3.2 (2.08)	**	2.8 (1.82)	2.6 (1.69)	
Packaging with smells	3.8 (1.89)	4.0 (1.91)	3.8 (1.88)		3.8 (1.87)	4.1 (2.03)		3.7 (1.83)	3.9 (1.93)	
Packaging that incorporates new technologies that improve the product experience (virtual reality, conductive ink, QR)	4.0 (1.85)	4.2 (1.78)	3.9 (1.88)	*	4.0 (1.85)	4.0 (1.96)		4.1 (1.82)	3.9 (1.87)	
Packaging that brings more interaction with the product	4.7 (1.71)	4.9 (1.68)	4.6 (1.72)	*	4.7 (1.71)	4.6 1.75)		4.5 (1.66)	4.8 (1.73)	**
Packaging that informs whether the product is at the ideal consumption temperature	5.5 (1.40)	5.5 (1.49)	5.4 (1.41)		5.5 (1.49)	5.4 (1.34)		5.3 (1.40)	5.5 (1.40)	*
Frozen or fresh food packaging with indicators that guarantee that the product has been maintained at the correct temperature	5.9 (1.24)	5.9 (1.20)	5.8 (1.25)		5.9 (1.23)	5.8 (1.30)		5.7 (1.27)	5.9 (1.21)	*
**Total**	**4.4**	**4.6**	**4.4**		**4.4**	**4.5**		**4.3**	**4.4**	

Participants indicated their degree of agreement using a 7-point Likert scale (1, definitely not; 7, definitely yes). *p* ≤ 0.05 * and *p* ≤ 0.01 **.

## Data Availability

The data presented in this study are partly available online at http://www.actinpak.eu/wp-content/uploads/2018/11/Roadmap_WG2_interactive.pdf (accessed on 18 February 2021). Tiekstra, S.; Lahti, J.; Buntinx, M. ROADMAPS Socio-Economic; 2019; pp. 1–10.

## Supplementary Materials

A bigger and more detailed version of Figure 6, the socioeconomic roadmap composed by Working Group 2 of COST Action FP1405 can be downloaded at http://www.actinpak.eu/roadmap-wg2/.
